# Exendin-4 Reduces Ischemic Brain Injury in Normal and Aged Type 2 Diabetic Mice and Promotes Microglial M2 Polarization

**DOI:** 10.1371/journal.pone.0103114

**Published:** 2014-08-07

**Authors:** Vladimer Darsalia, Sansan Hua, Martin Larsson, Carina Mallard, David Nathanson, Thomas Nyström, Åke Sjöholm, Maria E. Johansson, Cesare Patrone

**Affiliations:** 1 Karolinska Institutet, Department of Clinical Science and Education, Södersjukhuset, Internal Medicine, Stockholm, Sweden; 2 Department of Physiology, Institute of Neuroscience and Physiology, The Sahlgrenska Academy, University of Gothenburg, Gothenburg, Sweden; 3 Department of Biochemistry and Molecular Biology, College of Medicine, University of South Alabama, Mobile, Alabama, United States of America; 4 Department of Internal Medicine, Diabetes Research Unit, Södertälje Hospital, Södertälje, Sweden; University of Lancaster, United Kingdom

## Abstract

Exendin-4 is a glucagon-like receptor 1 agonist clinically used against type 2 diabetes that has also shown neuroprotective effects in experimental stroke models. However, while the neuroprotective efficacy of Exendin-4 has been thoroughly investigated if the pharmacological treatment starts before stroke, the therapeutic potential of the Exendin-4 if the treatment starts acutely after stroke has not been clearly determined. Further, a comparison of the neuroprotective efficacy in normal and aged diabetic mice has not been performed. Finally, the cellular mechanisms behind the efficacy of Exendin-4 have been only partially studied. The main objective of this study was to determine the neuroprotective efficacy of Exendin-4 in normal and aged type 2 diabetic mice if the treatment started after stroke in a clinically relevant setting. Furthermore we characterized the Exendin-4 effects on stroke-induced neuroinflammation. Two-month-old healthy and 14-month-old type 2 diabetic/obese mice were subjected to middle cerebral artery occlusion. 5 or 50 µg/kg Exendin-4 was administered intraperitoneally at 1.5, 3 or 4.5 hours thereafter. The treatment was continued (0.2 µg/kg/day) for 1 week. The neuroprotective efficacy was assessed by stroke volume measurement and stereological counting of NeuN-positive neurons. Neuroinflammation was determined by gene expression analysis of M1/M2 microglia subtypes and pro-inflammatory cytokines. We show neuroprotective efficacy of 50 µg/kg Exendin-4 at 1.5 and 3 hours after stroke in both young healthy and aged diabetic/obese mice. The 5 µg/kg dose was neuroprotective at 1.5 hour only. Proinflammatory markers and M1 phenotype were not impacted by Exendin-4 treatment while M2 markers were significantly up regulated. Our results support the use of Exendin-4 to reduce stroke-damage in the prehospital/early hospitalization setting irrespectively of age/diabetes. The results indicate the polarization of microglia/macrophages towards the M2 reparative phenotype as a potential mechanism of neuroprotection.

## Introduction

Stroke is one of the major causes of death and adult disability. The risk of stroke dramatically increases along aging and three-quarters of all strokes occur in people over the age of 65 (reviewed in [1]). Type 2 diabetes (T2D) increases the risk of stroke 2-6-fold when compared with non-diabetic individuals. In addition, T2D doubles the risk of stroke recurrence and increases mortality (reviewed in [2]).

Neuroprotective strategies aimed at decreasing brain damage after stroke have failed to be translated into the clinical setting along the past decades [3] [4], [5]. Today, tissue plasminogen activator (tPA) is the only established pharmacological treatment that restores brain reperfusion [6]. However, only a low rate of patients receives tPA due to delayed hospitalization and side effects (*e.g*. intracerebral hemorrhage) [6], [7]. As a consequence, there is a large un-met medical need for novel stroke therapies.

Glucagon-like peptide-1 (GLP-1) is an incretin hormone secreted from enteroendocrine L-cells following a meal [8]. GLP-1 enhances glucose-dependent insulin secretion *via* a specific G-protein-coupled GLP-1 receptor (GLP-1R) [8]. However, GLP-1 has a very short half-life in the peripheral blood mainly due to rapid enzymatic degradation [9]. Exendin-4 (Ex-4) is a synthetic form of GLP-1, which is resistant to degradation. For these properties, it has been developed in clinical use for the treatment of T2D [10]. Besides its anti-diabetic properties, Ex-4 has been shown to cross the blood brain barrier (BBB) [11] in dose-dependent manner and preclinical work supports a neuroprotective role of Ex-4 in animal models of neurological disorders (reviewed in [12]–[14]). With regard to stroke, intracerebral administration of Ex-4 before stroke resulted in neuroprotection [15]. Furthermore, others and we have shown that peripheral administration of Ex-4 before stroke induces neuroprotection [16]–[19]_ENREF_21. Thus, strong experimental evidence indicates the potential use of Ex-4 for the treatment of stroke in T2D patients or individuals at high risk to suffer from a stroke (*e.g*. pretreatment strategies). However, whether Ex-4 is neuroprotective when administered acutely after stroke in a clinical relevant setting e.g. few hours after stroke, has not been thoroughly investigated. Furthermore, whether T2D and aging induce a differential neuroprotective response to Ex-4 has not been studied. Finally the molecular mechanisms at the basis of Ex-4 neuroprotection have not been characterized.

Inflammation is a key mediator in brain damage after stroke [20]–[23]. Microglia, the resident macrophage of the brain, plays a central role in brain inflammation. Microglia/macrophages are highly dynamic cells that can adapt different phenotypes depending on the microenvironment, *i.e*. the classical pro-inflammatory M1 phenotype and the wound-healing reparative M2 phenotype respectively [24]. After stroke there is an acute/early polarisation towards the reparative M2 phenotype, however, over time there is an increased polarisation towards the pro-inflamamtory M1 phenotype [25].

The aim of this study was to determine the potential efficacy of Ex-4 against stroke in a clinically relevant post-stroke setting, in both young and aged T2D/obese mice. Furthermore, we determined whether Ex-4 has an effect on inflammatory markers and the microglial phenotype.

## Materials and Methods

### Animals and experimental groups

One hundred and twenty eight male C57Bl mice (Nova-SCB, Stockholm, Sweden) were used in the experiments.

#### Study 1

Thirty one 2-month old mice received 5 µg/kg Ex-4 intraperitoneally (i.p.) at 1.5 (n = 10) or 3 hours (n = 10) after transient middle cerebral artery occlusion (MCAO). Control mice (n = 11) were given vehicle injection 1.5 hours after MCAO. The treatment continued for 7 days with the dose of 0.2 µg/kg daily until sacrifice.

#### Study 2

Forty seven 2-month-old mice were treated with 50 µg/kg Ex-4 e i.p. at 1.5 (n = 13), 3 (n = 13) or 4.5 (n = 7) hours after MCAO. Control mice (n = 14) were given vehicle injection 1.5 hours after MCAO. The treatment continued for 7 days as in Study 1 (0.2 µg/kg Ex-4) until sacrifice.

#### Study 3

Twenty seven 2-month-old mice were exposed to high-fat diet (HFD) (Research Diets, Inc., New Brunswick, NJ) for 12 months to induce T2D/obesity. Body weight and blood glucose levels were monitored throughout the experiment (see Fig. S1 in file S1). At the end of the diet period, mice were subjected to stroke and received 50 µg/kg Ex-4 i.p. at 1.5 (n = 9) or 3 hours (n = 9) after MCAO. Control mice (n = 9) were given vehicle injection 1.5 hours after MCAO. The treatment continued for 7 days as in Studies 1–2 until sacrifice.

#### Study 4 (inflammation study)

Twenty-three 2-month old mice were treated with 50 µg/kg Ex-4 (n = 12) or vehicle (n = 11) i.p. at 1.5 hours after MCAO. The treatment continued for 3 days with the Ex-4 dose of 0.2 µg/kg daily until sacrifice.

All experiments were conducted according to the "Guide for the Care and Use of Laboratory Animals" published by U.S. National Institutes of Health (NIH publication # 85–23, revised 1985) and approved by the regional ethics committee (Stockholm Södra Djurförsöketiska Nämnd) for animal experimentation (applications S17–10 and S7–13).

### Transient middle cerebral artery occlusion

The intraluminal filament model of focal ischemia was used [26]. Briefly, a silicon-coated monofilament was inserted into internal carotid artery until the origin of MCA was occluded. The filament was withdrawn after 30 minutes of occlusion. For all experimental details of the MCAO method, see File S1.

### Immunohistochemistry, infarct volume measurement and neuronal quantifications

**Figure 1:**
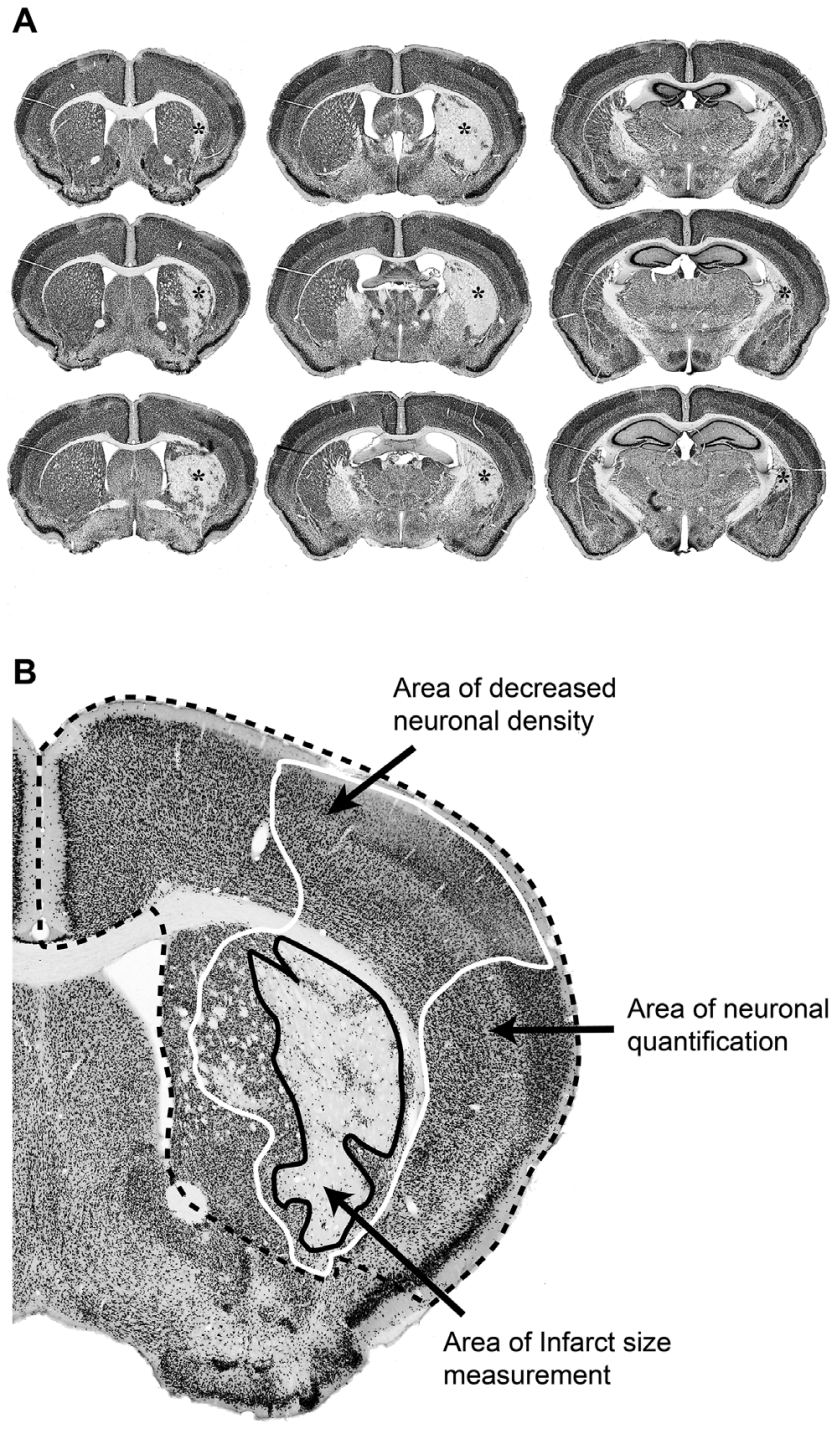
Stroke damage analyses. A. Representative photomicrographs of brain sections (NeuN immunostained) used in quantitative analyses. B. Representative image of stroke-damaged brain. Solid black line denotes the area of the infarct size/stroke volume measurement. Solid white line denotes the area of decreased neuronal density after MCAO. Dashed black line denotes the area of neuronal quantification by stereology methods.

Brains were fixed with trans-cardial 4% paraformaldehyde perfusion, sectioned using sliding microtome and immunohistochemically labeled for neuronal nuclear marker NeuN using peroxidase substrate method. Nine consecutive 40 µm-thick coronal sections spaced at 320 µm intervals along the rostra-caudal axis starting approximately at 1.5 mm from bregma were used for the stroke volume measurement and stereological counting of surviving neurons (Fig. 1A). For details, see File S1.

### Microglial cell culture and multiplex analysis

Primary microglia-enriched cultures were prepared from whole brains of 2–3 day-old mice. See File S1 for details.

### RNA extraction, cDNA synthesis and gene expression analysis

Frozen brain samples were homogenized with cold RNAse free PBS. RNA was extracted by using the RNAeasy Lipid Tissue Mini Kit (Qiagen GmbH, Hilden, Germany) according to the manufacturer's protocol. See File S1 for details.

### Statistical analyses

In studies 1–3 and microglia *in vitro* studies, statistical analyses were performed using Student's unpaired t-test or one-way analysis of variance (ANOVA), followed by Tukeys multiple comparisons test. Inflammatory and microglia markers in study 4 were analyzed using Kruskal-Wallis one-way analysis of variance followed by Dunn's multiple comparison test (Prism, GraphPad Prism 5, GraphPad Software, Inc, CA). Differences between groups were considered statistically significant when *P*<0.05. Data are presented as means ± SEM.

## Results

### Ex-4 treatment reduces brain damage in a dose-dependent manner when given up to 3 hours after stroke onset

**Figure 2:**
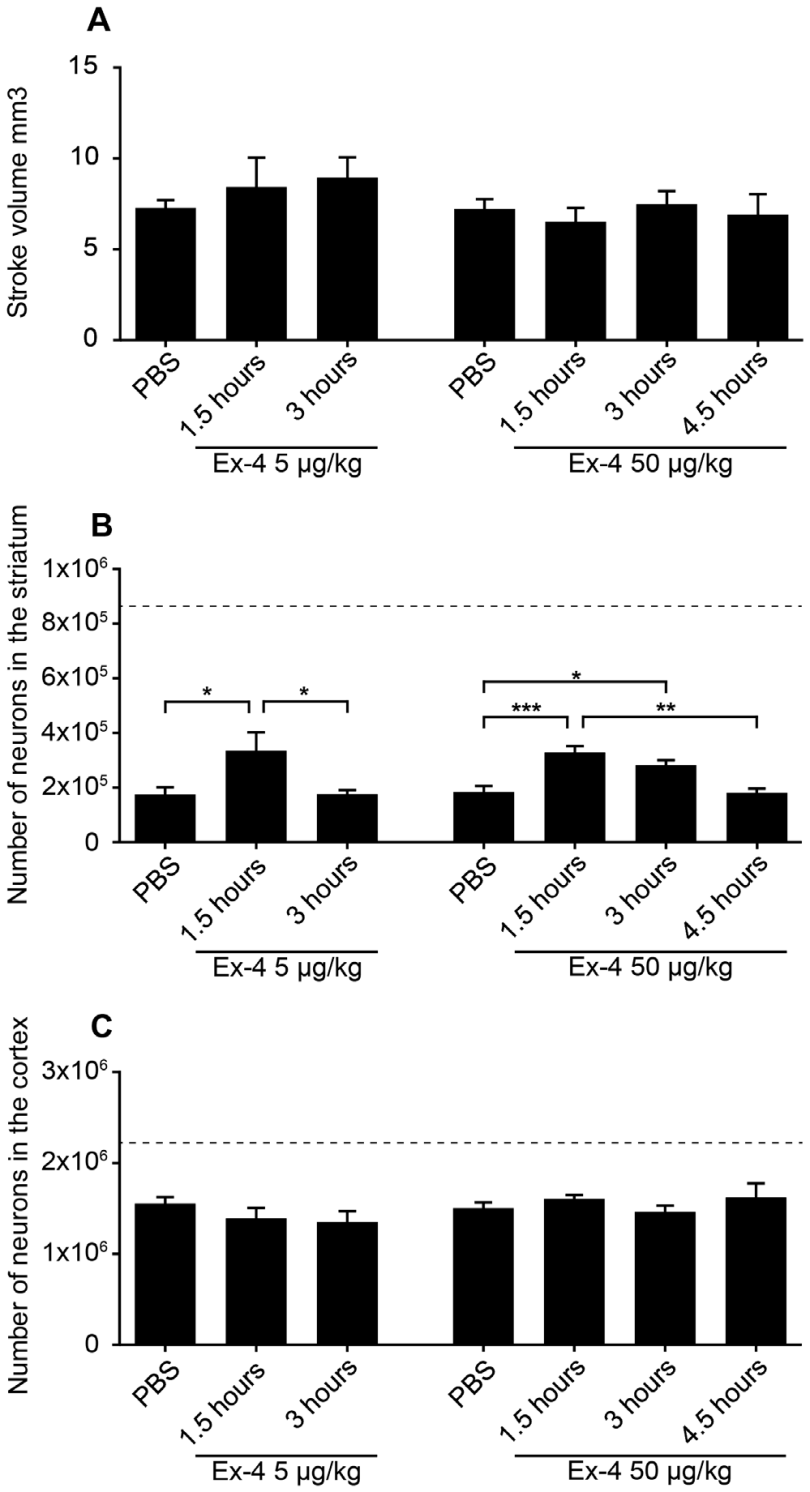
Neuroprotective effects of Ex-4 in normal, adult mice (Studies 1–2). Stroke volume at one week after 5 µg/kg or 50 µg/kg of Ex-4 administration (A). Number of surviving neurons in striatum (B) and cortex (C) at one week after 5 µg/kg or 50 µg/kg Ex-4 administration at 1.5, 3 and 4.5 hours after MCAO. The dashed lines on B, C indicate the number of neurons in the contralateral, corresponding intact brain areas. Data are presented as means ± SEM. *, ** and *** denote p<0.05, p<0.01 and p<0.001 respectively.

In Study 1, we determined the potential neuroprotective efficacy of one *bolus* of 5 µg/kg Ex-4 that was administered 1.5 or 3 hours after MCAO in adult healthy mice. The treatment was continued with 0.2 µg/kg/day Ex-4 for 1 week until sacrifice. The results show that the volume of the ischemic damage was similar in all animals irrespective of Ex-4 administration time (Fig. 2 A). However, more precise evaluation of the number of surviving neurons by stereological counting revealed a significant neuroprotective effect of Ex-4 treatment at 1.5 hours, but not at 3 hours after MCAO (Fig. 2 B). The protective effect was localized specifically in the striatum but not in cortex (Fig. 2 B, C).

In Study 2, we increased the dose of Ex-4 to one *bolus* of 50 µg/kg at 1.5, 3 or 4.5 hours after MCAO. Similarly to the first experiment, the treatment was continued for 1 week with 0.2 µg/kg/day. The results show that the stroke volume was not affected (Fig. 2 A). However the neuronal loss was significantly reduced at both 1.5 and 3 hours after- MCAO by the Ex-4 treatment (Fig. 2 B). The protective effect of Ex-4 was lost when the treatment was given at 4.5 hours after MCAO (Fig. 2 B). As in Study 1, the protective effect of Ex-4 occurred in striatum but not in cortex (Fig. 2 B, C). To show the impact of the stroke induced by MCAO on neuronal numbers in the striatum and cortex, the number of neurons in the non-stroke contralateral striatum and cortex are represented by the dashed line on Fig 2 B, C.

### Ex-4 treatment is neuroprotective up to 3 hours after MCAO in aged T2D/obese mice

**Figure 3:**
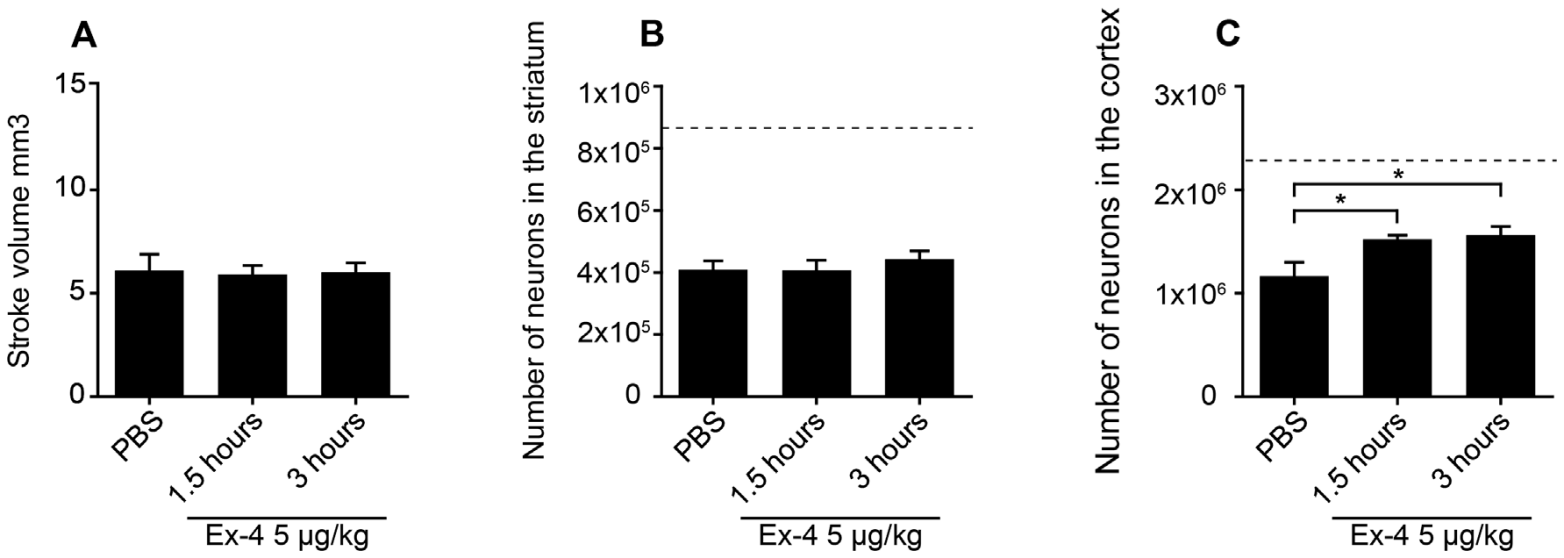
Neuroprotective effects of Ex-4 in aged, diabetic/obese mice (Study 3). Stroke volume at one week after 50 µg/kg (A) Ex-4 administration. Number of surviving neurons in the striatum (B) and cortex (C) at one week after 50 µg/kg Ex-4 administration at 1.5 and 3 hours after MCAO. The dashed lines on B and C indicate the number of neurons in the contralateral corresponding intact brain areas. Data are presented as means ± SEM. * denotes p<0.05.

In Study 3 we determined the potential efficacy of Ex-4 in aged 14-month-old T2D/obese mice. A *bolus* of 50 µg/kg Ex-4 was administered at 1.5 or 3 hours after MCAO. The Ex-4 treatment was continued at 0.2 µg/kg/day for 1 week until sacrifice as in studies 1–2. Similarly to 2-month-old healthy mice, Ex-4 showed neuroprotective efficacy at both 1.5 and 3 hours after- MCAO administration (Fig. 3 C). However, in contrast to the previous experiments, the neuroprotective effect of Ex-4 was localized in the cerebral cortex and not in striatum (Fig. 3 B, C). To show the impact of MCAO on neuronal numbers in the striatum and cortex, the number of neurons in the non-stroke contralateral striatum and cortex are represented by the dashed line on Fig 3 B, C.

### The Ex-4 neuroprotective effect correlates with M2 microglial phenotype

**Figure 4:**
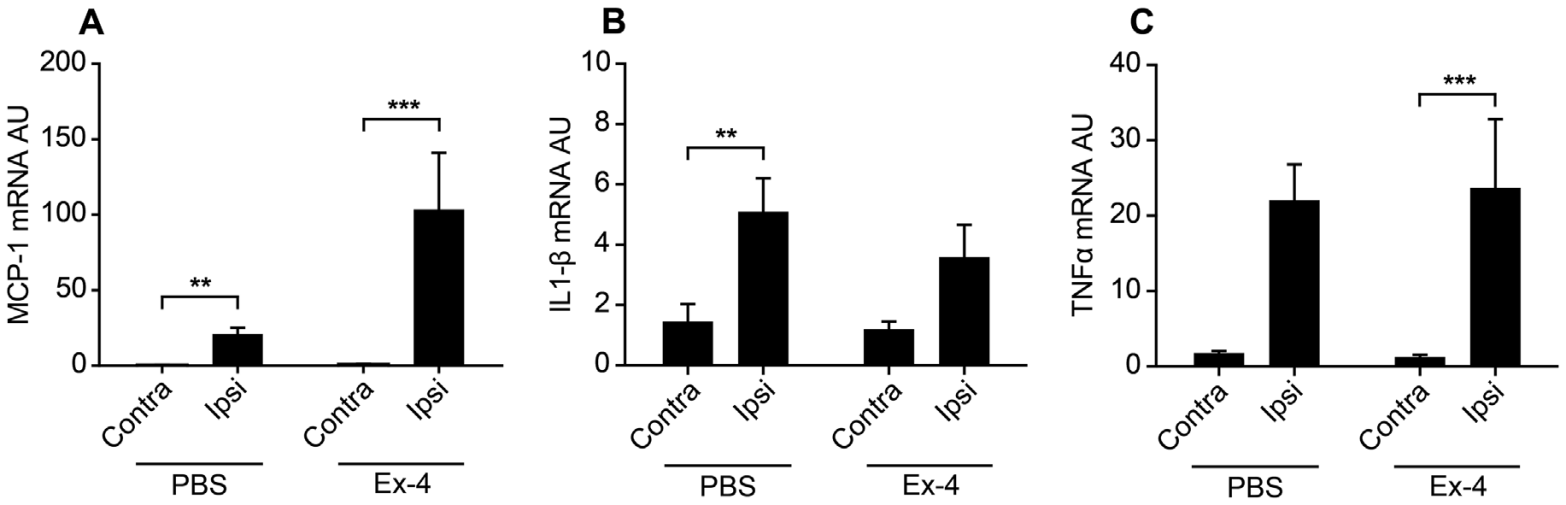
Stroke increase gene expression of pro-inflammatory cytokines (Study 4). Gene expression of pro-inflammatory cytokines MCP-1 (A), IL-1β (B) and TNFα (C) was analyzed by real-time PCR in brain homogenate from mice 3 days after MCAO. Gene expression was normalized to YWHAZ and expressed as arbitrary units (AU). Data are presented as means ± SEM. ** and *** denote p<0.01 and p<0.001 respectively.

In Study 4 we determined the gene expression of pro-inflammatory markers MCP-1, IL-1β and TNFα with and without Ex-4 treatment following MCAO. Pro-inflammatory cytokines MCP-1 and IL-1β were significantly up regulated in the injured hemisphere following MCAO (Fig. 4). A similar pattern was seen for TNFα albeit it did not reach statistical significance. Ex-4 treatment did not alter this pro-inflammatory pattern although a not statistically significant trend towards the enhancement of MCP-1 levels was observed in the ipsilateral hemisphere of Ex-4-treated mice as compared to vehicle-treated group. In support, lipopolysaccharide (LPS)-induced IL-1β and MCP-1 release from microglia cell cultures was also not affected by Ex-4 treatment (Fig. S2 in file S1) nor were any of the other cytokines or chemokines measured in the multiplex assay (data not shown).

**Figure 5:**
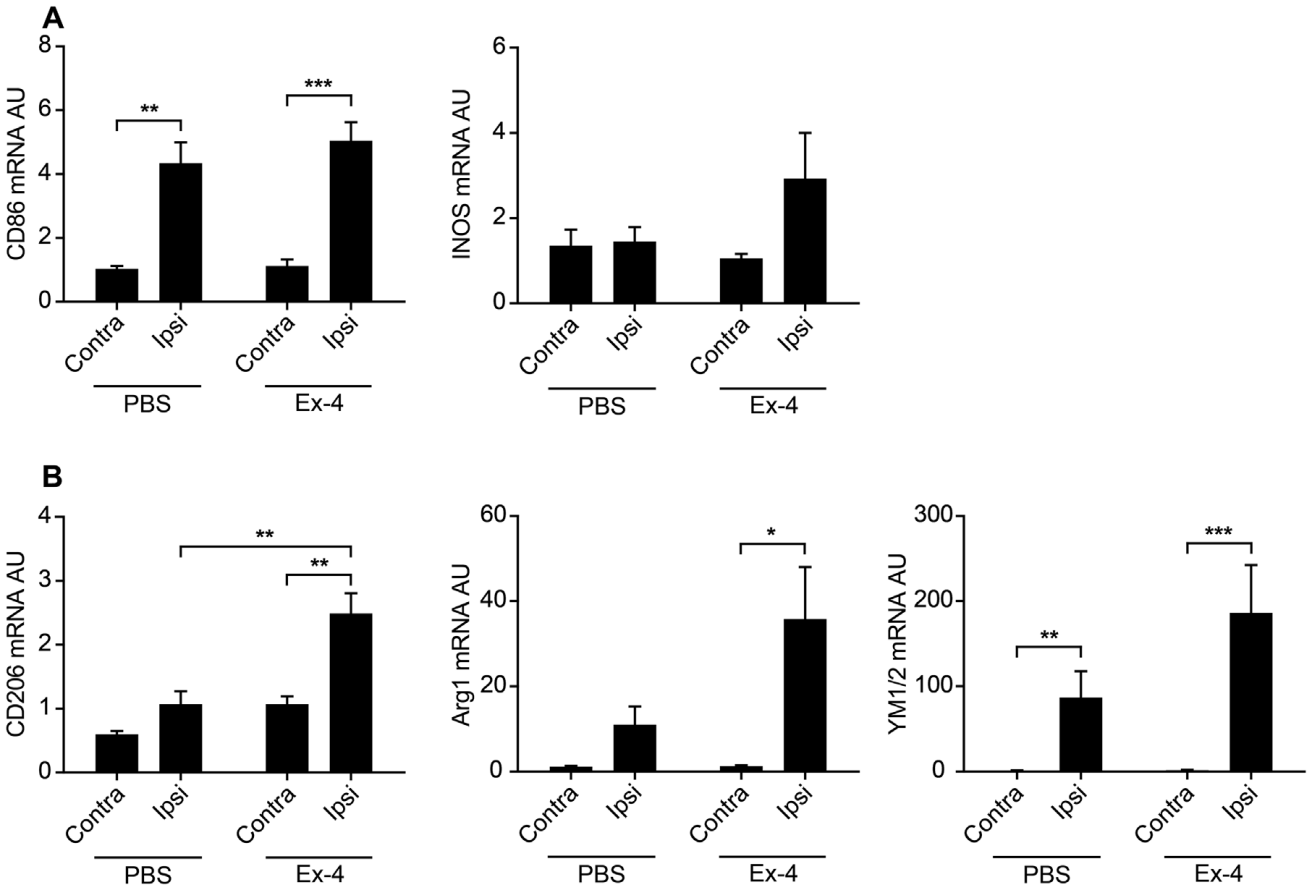
Ex-4 treatment polarizes microglia towards a reparative M2 phenotype (Study 4). Microglial phenotype was determined by real-time PCR analysis of M1 markers CD86 and, iNOS (A), and M2 markers CD206, Arginase1 (Arg1) and YM1/2 (B) 3 days after MCAO. Gene expression was normalized to YWHAZ and expressed as arbitrary units (AU). Data are presented as means ± SEM. *, ** and *** denote p<0.05, p<0.01 and p<0.001 respectively.

Microglia can polarize into different phenotypes depending on the microenvironment (see introduction). To address the microglial phenotype, we quantified gene expression of M1 markers iNOS and CD86, and M2 markers CD206, Arg1 an YM1/2, respectively. Ischemia significantly increased M1 marker CD86 in the injured ipsilateral hemisphere in both control and Ex-4-treated mice (Fig. 5 A). However, expression of the M1 marker iNOS was not altered (Fig. 5 A). The quantitative analysis of M2 markers, revealed that CD206 and Arg1 were not significantly affected by the ischemic brain damage, whereas YM1/2 was significantly up regulated in the injured hemisphere (Fig. 5 B). Ex-4 treatment significantly up regulated M2 markers CD206, Arg1 and YM1/2 in the injured hemisphere compared to the non-injured hemisphere (Fig. 5 B). Furthermore, CD206 expression in the injured, Ex-4-treated hemisphere was significantly up regulated compared to the injured control hemisphere (Fig. 5 B). A similar pattern was also seen for Arg1 and YM1/2(Figure 4D–E). However this did not reach statistical significance.

## Discussion

Several studies have shown that Ex-4 mediates neuroprotection in animal models of stroke. These studies have demonstrated proof-of-concept for Ex-4-mediated neuroprotection against ischemic brain damage [15]–[19]. However, in these studies the neuroprotective efficacy was achieved either by using strategies based on pretreatment [16], [17], or by using experimental paradigms difficult to be translated to the clinical reality, *e.g*. intracerebral administration [15], [18]. Thus, the potential neuroprotective efficacy of Ex-4 if the pharmacological treatment starts acutely after MCAO, which is a clinically relevant setting, has not been extensively investigated. In clinical stroke, the intervention time (rTPA) usually falls between 0.5 and 4 hours and the speed of intervention has been positively correlated with favorable stroke outcome [27]–[29]. The goal of our study was to evaluate the efficacy time-window for Ex-4-mediated neuroprotection after the stroke onset; hence we treated MCAO-subjected mice with Ex-4 at 1.5, 3 and 4.5 hours after MCAO. Interestingly, in one study, Teramoto et al, showed that Ex-4 was neuroprotective if given 1 hour after MCAO (stroke volume was measured 24 hours after stroke [19]). Under these conditions, the neuroprotective effect was lost when Ex-4 was administered at 3 hours after MCAO. It has to be noted that the Ex-4 dose used in this study (approx. 400 µg/kg) was far higher than the used clinical dose of Ex-4 to treat T2D patients (0.1–0.2 µg/kg). In our study, we show that Ex-4 mediates neuroprotection in both healthy and T2D/obese mice by giving a *bolus* of 50 µg/kg Ex-4 from 1.5 or 3 hours after MCAO and by continuing with the treatment for one week with lower (clinical) dose of the drug (0.2 µg/kg). Furthermore, in normal healthy mice we show that the lower dose of 5 *µ*g/kg was still efficacious at 1.5 hour after MCAO, although the effect was lost at 3 hours. The discrepancy between our results and the study by Teramoto et al at 3 hours are likely due to different methods of stroke evaluation. The stroke volume measurement (used by Teramoto et al) is a macro readout and does not account for decreased neuronal density in peri-infarct areas while neuronal count (used in our study) evaluates the whole brain structure (Figure 1B). Indeed, the stroke volume measurements in both studies did not detect neuroprotection by Ex-4 at 3 hours after MCAO. In contrast to previously published studies where Ex-4 was given before or at the time of stroke, the Ex-4-mediated reduction of the infarct volume in the present study was marginal and the efficacy was mainly localized in the surrounding *penumbra* tissue. These results are not surprising, considering that initial excitotoxic cell death (the biggest contributor of tissue damage) inside the ischemic core occurs within minutes after stroke. For any neuroprotective therapy that is initiated few hours after stroke the main target would be a relatively smaller population of neurons localized within the ischemic penumbra. These neurons are under the threat, but it is still possible to rescue them with a proper intervention [30].

The most efficacious dose of Ex-4 in our study was a bolus of 50 µg/kg followed by daily injections of 0.2 µg/kg. Decreasing the first bolus injection from 50 µg/kg to 5 µg/kg resulted already in diminished efficacy. Since 0.2 µg/kg is the clinically used dose to treat T2D clinically, it is unlikely that this dose will be within the efficacy range for mediating neuroprotection after stroke. As a consequence, a new evaluation for the safety of Ex-4 will be needed to determine the safety/feasibility of higher Ex-4 doses to stroke patients if such therapy will become a clinical option

Moreover in our results we clearly see the correlation between the efficacy, the dose and the intervention time.

The major obstacles for the development of neuroprotective stroke therapies depend upon how quickly neurons die: “time is brain”. Because of this, several preclinically successful candidate drugs (used before/few minutes after stroke) have failed in clinical trials once administered to patients several hours after stroke [3], [31], [32]. Although it is difficult to exactly translate the neuroprotective window of Ex-4 from mouse to human, our results might have clinical relevance. However, since the protective effect of Ex-4 was lost at 4.5 hours, we foresee the potential use of Ex-4 for the treatment of stroke as early as possible after the ischemic event, possibly already during the emergency transport. Ex-4 is an antidiabetic drug that has been reported to show limited side effects [33] and it is formulated for subcutaneous self-injections. Thus, at least in theory, stroke patients should be able to receive this treatment with minimal risks before hospitalization.

Many stroke patients have comorbidities and conditions such as advanced age, hypertension, obesity and T2D. Often preclinical efficacy studies are not performed in animals with such comorbidities and this is likely another reason, in addition to time, why preclinical neuroprotective strategies tested in young healthy animals have failed in the clinic [31], [32]. To determine whether both aging and T2D could have an impact on the Ex-4 neuroprotective efficacy, we performed stroke experiments in aged and T2D/obese mice. Our results showed no decrease in efficacy by Ex-4 in aged T2D/obese *versus* adult healthy mice, suggesting that this therapy has the potential to be extended to also aged and diabetic patients. Although Ex-4 was efficacious in both normal and aged T2D/obese mice, we observed a differential neuroprotective effect at the anatomical level between the two groups, *e.g*. striatal in normal while cortical in T2D/obese mice. These observations are difficult to interpret and are likely the results of the combination of the effects of aging and T2D, which could alter the outcome of MCAO. Indeed, in the striatum of young adult mice, the ischemic damage was more pronounced as compared to aged/T2D mice (Fig 2 B and fig 3 B, control groups). On the contrary, in aged/T2D mice the cortical damage was more severe as compared to adult healthy mice (Fig 2 C and Fig 3 C, control groups). It is known, that diabetes can induce alterations in the cerebral blood flow on microvascular level [34], [35] and such alterations could be behind the differential response to MCAO between adult/healthy and aged/T2D mice. Although it is only speculative, it is possible that a larger stroke in either brain areas could produce a larger *penumbra* region in which the effect of Ex-4 could be more readily detected as opposed to smaller ischemic damaged areas. To our knowledge, this is the first study that has employed both aged and T2D/obese mice to study stroke. Thus, no comparison with previous work could be done. Overall, the differential stroke outcome between normal and T2D/obese mice underscores the importance to study stroke and potential therapies by using stroke models with comorbidities as recommended by the STAIR group [36]. Parallel studies in normal and T2D rodents might reveal important information to understand both the dynamics and the mechanisms of stroke, but also to choose the most appropriate therapy.

The ultimate goal of any stroke intervention therapy is to improve the functional outcome. However, the available mouse stroke models present significant challenges for accurate functional evaluation and current tests can unlikely detect neurological function with enough sensitivity to identify the subtle effects of neuroprotective treatments [37]. Li et al have reported that several common functional tests failed to detect differences even between sham and stroke-subjected mice several days after 90 minutes MCAO. Furthermore, at 2 weeks after stroke, even the most accurate tests at their disposal failed to recognize functional differences between untreated mice and mice treated with estrogen (well recognized neuroprotective substance) [37]. We have used 30 minutes of MCAO in our study and Ex-4 treatment resulted in ≈25% increase in neuronal survival. Thus, we can only speculate the functional benefits of such neuroprotection.

Despite the fact that neuroprotection by Ex-4 has been shown in animal models of stroke, the mechanisms behind this neuroprotection remain elusive. In addition to direct neuroprotective effects also supported by GLP-1R expression in neurons [38], the regulation of inflammation has been suggested as one potential mechanism since Ex-4 decreases the number of microglia after stroke [16], [18], [19]. Furthermore, it has been shown that GLP-1 treatment decreases LPS-induced inflammation in astrocytes *in vitro* [39]. Interestingly, the neuroprotective effect of Ex-4 in our stroke model is not correlated to decreased inflammation, since Ex-4 treatment did not alter gene expression of pro-inflammatory cytokines. This observation was unexpected. However it is most likely related to the differences in study design between this current study and the ones previously published [16], [18], [19] where the Ex-4 treatment was initiated before or at the time of stroke and the effect on stroke size was dramatic. It is plausible, that the previously observed anti-inflammatory effects (reduced microglia numbers) by Ex-4 were secondary to the reduced injury size, rather than being a direct consequence of anti-inflammatory effects of Ex-4. Our *in vitro* data further support this by showing no effect of Ex-4 on LPS-induced inflammation in cultured microglia. Similar to that shown after both spinal cord injury [40] and focal cerebral ischemia [25], we found an increased expression of both M1 and M2 markers 3 days after MCAO. Administration of Ex-4 had no effect on the M1 markers. In contrast, Ex-4 increased the expression of M2 markers after MCAO. It has been suggested that the microglial M2 phenotype has a reparative function and could promote CNS repair [40]. Further, we have previously shown that increased vulnerability after brain injury was associated with a decrease in reparative M2 microglia [41]. The increased expression of M2 markers by Ex-4 indicates that Ex-4 enhances the polarization towards the reparative M2 phenotype, suggesting a novel mechanism at the basis of the neuroprotective efficacy mediated by Ex-4. This mechanism based on M2 repair could also contribute to the larger therapeutic window (till 3.5 hours) observed in our study in comparison to that reported by Taramoto et al [19] (1 hour). In the latter study, the mice received Ex-4 only once before being sacrificed 24 hours thereafter. *Vice versa*, in our study we continued with daily treatments of Ex-4 for several days, thus potentially maximizing the Ex-4-mediated M2 reparative phenotype.

In conclusion, we show that Ex-4 mediates neuroprotection against stroke in normal and aged T2D/obese mice. The results were achieved by using a preclinical experimental paradigm with potential relevance for the treatment of stroke patients in the prehospital or early hospitalization settings. The results also suggest that one of the contributing mechanisms at the basis of Ex-4 neuroprotection may be enhancing the reparative M2 phenotype.

## Supporting Information

File S1 Contains the following files: **Figure S1**. The HFD regime leads to increased body weight gain and increased fasting and fed blood glucose levels. Body weight in 2-month-old healthy mice or after 12 months of HFD (A). Fasting blood glucose levels in 12-month-old HFD-fed and 2-month-old healthy mice are shown (B). Fed blood glucose levels in HFD-fed mice (14 months old) and in 2-month-old healthy mice at the time of the MCAO surgery (C). Data are presented as means ± SEM. **** denotes p<0.0001. **Figure S2**. Ex-4 treatment does not change inflammatory markers in microglia cultures. Microglial cell cultures were stimulated with the pro-inflammatory mediator LPS (10 ng/ml) for 24 h with or without Ex-4 (40 ng/ml). IL-1β (A) and MCP-1 (B) were measured in the microglia media, Graphs represent data from 3 independent experiments. Data are presented as means ± SEM. ***p<0.001.
(DOCX)
