# Supplementary material for: Exendin-4 Reduces Ischemic Brain Injury in Normal and Aged Type 2 Diabetic Mice and Promotes Microglial M2 Polarization
Source: PLoS One. 2014 Aug 7;9(8):e103114. doi: 10.1371/journal.pone.0103114 (PMC4125154; doi:10.1371/journal.pone.0103114)
Supplement: File S1 — Contains the following files: Figure S1. The HFD regime leads to increased body weight gain and increased fasting and fed blood glucose levels. Body weight in 2-month-old healthy mice or after 12 months of HFD (A). Fasting blood glucose levels in 12-month-old HFD-fed and 2-month-old healthy mice are shown (B). Fed blood glucose levels in HFD-fed mice (14 months old) and in 2-month-old healthy mice at the time of the MCAO surgery (C). Data are presented as means ± SEM. **** denotes p<0.0001. Figure S2. Ex-4 treatment does not change inflammatory markers in microglia cultures. Microglial cell cultures were stimulated with the pro-inflammatory mediator LPS (10 ng/ml) for 24 h with or without Ex-4 (40 ng/ml). IL-1β (A) and MCP-1 (B) were measured in the microglia media, Graphs represent data from 3 independent experiments. Data are presented as means ± SEM. ***p<0.001. (DOCX) [file pone.0103114.s001.docx]

**SUPPORTING INFORMATION FILE S1**

**Material and Methods**

***Measurements of body weight, fasting and fed blood glucose levels***

Body weight was monitored along the whole duration of the studies. Fasting blood glucose levels were measured after 12 months of HFD in T2D/obese mice. Fed blood glucose levels were assessed at the time of the MCAO when the mice were 14 months old. For comparison, the same measures were also performed in 2-month-old healthy mice (studies 1-2). Blood was drawn from a tail puncture, and glycemia was measured using a glucometer (One-Touch Ultra 2; LifeScan, Milpitas, CA)

***Microglial cell culture and Multiplex analysis***

Primary microglia-enriched cultures were prepared from whole brains of postnatal 2-3-day-old mice. Brains were extracted and homogenized. After homogenization, cells were seeded in Dulbecco’s modified Eagle’s medium (DMEM) with 20 % fetal bovine serum (FBS) and 1 % penicillin-streptomycin (Sigma Aldrich, Stockholm, Sweden) and cultured in 5 % CO_2_/95% air at 37°C. After 7 days *in vitro,* the medium was replaced with DMEM with 10% FBS / 1% penicillin-streptomycin. Confluence was achieved after 14 days *in vitro*. Enriched microglia cultures were obtained by using a reciprocating shaker at 250 rpm for 3 hours at 36°C. Microglia cells were pelleted via centrifugation at 250 g for 10 min, resuspended in DMEM with 2% FBS / 1% Penicillin-Streptomycin and 200,000-250,000 cells were plated/well. Following incubation for 24 h, cells were stimulated with LPS (10 ng/ml, List Biological Laboratories Inc, Campbell, CA) with or without Ex-4 (10nM/L). After 24 h incubation, supernatants were harvested and stored at -80°C for further analysis. Cytokines in microglial cell culture supernatants were measured by Bio-Plex Pro Assays according to the manufacturer’s protocol (Bio-Rad Laboratories, Inc., CA).

***RNA extraction, cDNA synthesis and gene expression analysis***

RNA quality and concentration were determined with gel electrophoresis (Experion, Bio-Rad Laboratories, CA) and NanoDrop analysis (NanoDrop Products, DE), respectively. All samples had an RIN value > 8. QuantiTect Reverse Transcription kit (Qiagen GmbH, Hilden, Germany) was used to synthesize first strand cDNA according to the manufacturer’s protocol.

Real-time PCR analysis was run on a LightCycler 480 (Roche Diagnostics GmbH, Mannheim, Germany) using the following cycling program; denaturation at 95°C for 10 minutes followed by 45 cycles of denaturation at 95°C for 15 seconds, and annealing /extension at 60°C for 4 seconds and 72°C for 10 seconds, respectively. Melting-curve analysis was performed to ensure that only one PCR product was obtained. PCR products were further validated on agarose gel. All samples were run in duplicates. Inter-sample differences were limited to 0.5 cycles and samples with >0.5 cycles difference were excluded from the analysis. The following primers were used; IL-1β (QT01048355), TNF-α (QT00104006), MCP-1 (ccl2, QT00167832), CD86 (QT01055250), iNOS (loc673161, QT01547980), CD206 (mrc1, QT00103012), YM1/2 (chi3l3, QT02241722), Arginase1 (arg1, QT00134288, all from Qiagen). The expression level of each target gene was normalized against the reference gene YWHAZ (tyrosine 3-monooxygenase/tryptophan 5-monooxygenase activation protein, QT00105350), calculated as 2-ΔΔCT, where ΔCT was the CT of the target gene after subtracting the CT value of the reference gene and ΔΔCT was the CT value corrected by the average CT of each group.

***Transient middle cerebral artery occlusion (tMCAO)***

Anesthesia was induced by 3% isoflurane and continued during surgery with 1.5% isoflurane using a snout mask. Briefly, the carotid arteries on the left side were exposed, the external carotid was ligated and temporary sutures were placed over the common carotid artery. Through a small incision in the external carotid artery, a 7-0 monofilament coated with silicone was advanced through the internal carotid artery until it blocked the origin of the middle cerebral artery. When the filament had been positioned, wounds were closed and the anesthesia was discontinued. After 30 minutes of occlusion, the mice were anesthetized again, the filament was withdrawn and the ligatures removed from the common carotid artery. Body temperature was maintained between 36 and 38°C with a heating lamp during surgery and ischemia, and the mice were then transferred to a heated box where they regained wakefulness and were kept there for 2 h. The surgeon performing the operation was blinded to the treatment groups.

***Immunocytochemistry***

Animals were deeply anesthetized with overdose of sodium pentobarbital and perfused transcardially with 4% paraformaldehyde. The brains were extracted, post-fixed in 4% paraformaldehyde overnight at 4°C and submersed in 20% sucrose in phosphate buffer until they sunk. 40 µm-thick coronal sections were cut using sliding microtome and stained as free-floating sections. The primary antibody anti-NeuN (1:100; Millipore, MA) was used to stain surviving neurons in striatum and cerebral cortex. Sections were incubated with the primary antibody for 36 hours at 4°C in phosphate buffer containing 3% normal horse serum and 0.25% Triton-X. Primary antibody was detected using biotin-conjugated anti mouse (Vector, CA) secondary antibody (1:200). For chromogenic visualization, avidin-boitin complex (ABC kit, Vector, CA) and diaminobenzidine were used. Brains with no visible stroke damage or visible hemorrhages were excluded from the analyses.

***Infarct volume measurement and cell quantifications***

For tissue damage evaluation, the NeuN-labeled tissue sections were displayed live on the computer monitor and the area of contralateral hemisphere and the area of the intact ipsilateral tissue were measured in every section containing stroke damage using NewCast (Visiopharm, Hoersholm, Denmark) software. To compensate for the stroke-induced morphological tissue changes, the infarct volume was calculated by subtracting the volume of remaining intact tissue in the ipsilateral hemisphere from the volume of the contralateral hemisphere. The number of neurons was quantified using the optical fractionator method[^1^](#_ENREF_1) as recently described in details[^2^](#_ENREF_2)^,^ [^3^](#_ENREF_3). Immunoreactive cells were counted using a computerized non-biased setup for stereology, driven by NewCast software.

**Results**

***Body weight and glycemia measures***

The results in Supplementary Figure 1A show that 12 months of HFD feeding (Study 3) resulted in a 3-fold increase in body weight in comparison with 2-month-old healthy mice (Studies 1-2). The results in Supplementary Figure 1B also show that fasting glucose levels after 12 months of HFD feeding were increased in comparison with healthy mice.. Fed blood glucose levels in HFD-fed mice were also significantly increased in comparison with healthy mice when measured at stroke time (Supplementary Fig. 1B-C).

***LPS-induced IL-1β and MCP-1 release from microglia cell cultures is not affected by Ex-4 treatment***

Cytokines in microglial cell culture supernatants were measured by Bio-Plex Pro Assays. The results show that LPS-induced IL-1β and MCP-1 release from microglia cell cultures was not affected by Ex-4 treatment (Fig. 2 below) nor were any of the other cytokines or chemokines measured in the multiplex assay (data not shown).

**Figure Legends**

***Figure S1. The HFD regime leads to increased body weight gain and increased fasting and fed blood glucose levels***

Body weight in 2-month-old healthy mice or after 12 months of HFD (A). Fasting blood glucose levels in 12-month-old HFD-fed and 2-month-old healthy mice are shown (B).  Fed blood glucose levels in HFD-fed mice (14 months old) and in 2-month-old healthy mice at the time of the MCAO surgery (C). Data are presented as means ± SEM. **** denotes p < 0.0001.

***Figure S2. Ex-4 treatment does not change inflammatory markers in microglia cultures.***

Microglial cell cultures were stimulated with the pro-inflammatory mediator LPS (10 ng/ml) for 24 h with or without Ex-4 (40 ng/ml). IL-1β (A) and MCP-1 (B) were measured in the microglia media, Graphs represent data from 3 independent experiments. Data are presented as means ± SEM. ***p<0.001

**Figures**

***Figure S1***


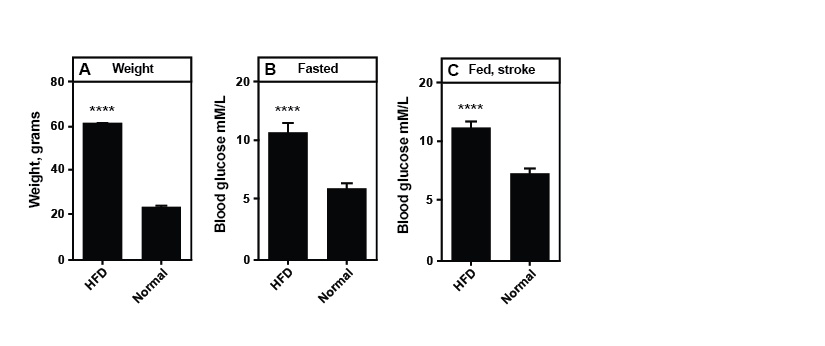


***Figure S2***

**References**

1. West MJ. Stereological methods for estimating the total number of neurons and synapses: Issues of precision and bias. *Trends in neurosciences*. 1999;22:51-61

2. Darsalia V, Mansouri S, Ortsater H, Olverling A, Nozadze N, Kappe C, et al. Glucagon-like peptide-1 receptor activation reduces ischaemic brain damage following stroke in type 2 diabetic rats. *Clin Sci (Lond)*. 2012;122:473-483

3. Darsalia V, Ortsater H, Olverling A, Darlof E, Wolbert P, Nystrom T, et al. The dpp-4 inhibitor linagliptin counteracts stroke in the normal and diabetic mouse brain: A comparison with glimepiride. *Diabetes*. 2013;62:1289-1296
